# Adolescents’ Filial Piety Attitudes in Relation to Their Perceived Parenting Styles: An Urban–Rural Comparative Longitudinal Study in China

**DOI:** 10.3389/fpsyg.2021.750751

**Published:** 2022-01-24

**Authors:** Li Lin, Qian Wang

**Affiliations:** ^1^School of Graduate Studies and Department of Applied Psychology, Lingnan University, Tuen Mun, Hong Kong SAR, China; ^2^Department of Applied Social Sciences, The Hong Kong Polytechnic University, Kowloon, Hong Kong SAR, China; ^3^Department of Psychology, The Chinese University of Hong Kong, Shatin, Hong Kong SAR, China

**Keywords:** bidirectional associations, filial piety attitudes, parenting styles, urban–rural comparison, Chinese adolescents

## Abstract

The Dual Filial Piety Model (i.e., the model of reciprocal and authoritarian filial piety) offers a universally applicable framework for understanding essential aspects of intergenerational relations across diverse cultural contexts. The current research aimed to examine two important issues concerning this model that have lacked investigation: the roles of parental socialization (i.e., authoritative and authoritarian parenting styles) and social ecologies (i.e., urban vs. rural settings that differ in levels of economic development and modernization) in the development of reciprocal and authoritarian filial piety attitudes. To this end, a two-wave short-term longitudinal survey study was conducted among 850 early adolescents residing in urban (*N* = 314, 49.4% females, mean age = 13.31 years) and rural China (*N* = 536, 45.3% females, mean age = 13.72 years), who completed questionnaires twice, 6 months apart, in the spring semester of grade 7 and the fall semester of grade 8. Multigroup path analyses revealed bidirectional associations over time between perceived parenting styles and adolescents’ filial piety attitudes, with both similarities and differences in these associations between urban and rural China. In both settings, perceived authoritative parenting predicted increased reciprocal filial piety 6 months later, whereas perceived authoritarian parenting predicted reduced reciprocal filial piety among urban (but not rural) adolescents over time. Moreover, in both settings, reciprocal filial piety predicted higher levels of perceived authoritative parenting and lower levels of perceived authoritarian parenting 6 months later, with the latter effect being stronger among urban (vs. rural) adolescents. Adolescents’ perceived parenting styles did not predict their authoritarian filial piety over time; however, authoritarian filial piety predicted higher levels of perceived authoritative parenting (but not perceived authoritarian parenting) 6 months later in both settings. The findings highlight the roles of transactional socialization processes between parents and youth as well as social ecologies in the development of filial piety, thus advancing the understanding of how the universal human motivations underlying filial piety may function developmentally across different socioeconomic and sociocultural settings.

## Introduction

Filial piety entails a set of psychological schemas of parent–child interactions, guiding intergenerational relationships across diverse family settings (Bedford and Yeh, 2019, 2021). Filial piety has been acknowledged to be a core pillar of Confucianism—a guiding ideology in Chinese culture (Ho, 1994)—and socializing children to endorse and practice filial piety is an important child-rearing goal for Chinese parents (Chao, 2000; Rao et al., 2003). However, rapid socioeconomic development in Chinese societies and the blending of cultures through globalization have presumably caused a transformation in Chinese people’s understanding and values concerning filial piety (Cheung and Kwan, 2009; Sun et al., 2019), resulting in changes in how children develop their filial piety in contemporary China.

According to the Dual Filial Piety Model (Yeh and Bedford, 2004; Bedford and Yeh, 2019), filial piety is comprised of two related but distinct types of psychological schemas: reciprocal filial piety, which entails a provision of support and care for parents due to affection and gratitude, and authoritarian filial piety, which entails child obedience and sacrifice due to parental authority or prescribed cultural norms. The Dual Filial Piety Model focuses on attitudes toward a set of filial behaviors, such as supporting parents when they are aged, rather than actual enactment of filial behaviors (Yeh and Bedford, 2004). Assessing attitudes enables us to understand individuals’ affections, values, or even behavioral tendencies toward a certain pattern of intergenerational relationships; however, cautions should be taken due to the potential mismatch between filial attitudes and behaviors (Chen et al., 2007). Research focusing on filial piety attitudes has found that reciprocal and authoritarian filial piety attitudes contribute to the quality of the parent–child relationship (e.g., Chen et al., 2016), children’s well-being (e.g., Sun et al., 2019), academic engagement (e.g., Chen, 2016), and psychosocial competences (e.g., Leung et al., 2010) in different ways. Yet, only minimal emerging research exists that examines how these two types of filial piety attitudes develop (e.g., Chen, 2014), leaving notable gaps in the extant literature. First, the current understanding is limited to “parent effects,” that is, how parenting styles or practices shape children’s filial piety attitudes (Chen, 2014; Chen et al., 2016). According to the transactional model of socialization (Sameroff and Mackenzie, 2003), socialization takes place through bidirectional influences between children and their environment (e.g., family), which points to a need to investigate “child effects,” that is, how children’s filial piety attitudes affect their parents’ parenting styles or practices. Filial piety prescribes how children should treat their parents, and thus, parents might adjust their parenting in response to the different filial piety attitudes of their offspring. Therefore, both parent effects (parenting→ children’s filial piety) and child effects (children’s filial piety→ parenting) should be considered in the study of the development of filial piety attitudes. Second, relatively little research has investigated the socio-ecologies in which the socialization of filial piety occurs, despite a call for understanding filial piety’s connection to individuals and the environment (Bedford and Yeh, 2021). Thus, it is crucial to investigate how socioeconomic and sociocultural changes (e.g., from rural to urban settings in China) make a difference in the socialization of filial piety.

To address these gaps, this study used a 6-month longitudinal design to examine bidirectional relationships between parenting styles and dual filial piety attitudes among Chinese early adolescents. We assessed attitudes rather than behaviors because young adolescents have not reached an age that can fulfill filial obligations, such as financially supporting their parents. Early adolescence was targeted because this is a critical period of identity formation that includes exploration and understanding of one’s role and duties in the family (Erikson, 1994). Furthermore, we compared these relationships between urban and rural China, as the uneven levels of socioeconomic development and modernization in urban and rural regions in China have presumably resulted in different understandings and values concerning filial piety (Chen and Li, 2012) and, in turn, may lead to different dynamics in the development of filial piety.

### The Dual Filial Piety Model

Filial piety is better understood in a dual model than as a single entity. Reciprocal filial piety is differentiated from authoritarian filial piety because the two are distinct in their motivation, manifestation, and implications for human adjustment (Yeh and Bedford, 2003). Reciprocal filial piety concerns children providing support and care to their parents, especially when their parents age, due to children’s gratitude for their parents’ nurturing and their intimate relationships with their parents. In contrast, authoritarian filial piety entails children showing unquestionable obedience toward their parents and protecting the family interests unconditionally, such as continuing the family lineage even at the cost of self-interests, because of parents’ authority status and prescribed cultural norms (Bedford and Yeh, 2019). Briefly stated, reciprocal filial piety emphasizes physical and emotional reciprocity between parents and children, while authoritarian filial piety stresses the family hierarchy and suppression of children’s desires for the sake of the family.

Mounting evidence has revealed different associations of reciprocal versus authoritarian filial piety with youth’s life satisfaction, psychosocial competence, and academic and behavioral adjustment. Reciprocal filial piety relates to higher levels of life satisfaction (e.g., Sun et al., 2019), academic achievement (e.g., Zhou et al., 2020) and interpersonal competence (Leung et al., 2010; Wang et al., 2021), and less cyberbullying perpetration (Wei and Liu, 2020), whereas authoritarian filial piety has opposite relationships with these outcomes. Given the well-documented conducive effects of reciprocal filial piety and the detrimental effects of authoritarian filial piety on youth development, it is imperative to examine how these two types of filial piety develop. Yet, to date, only a small body of research has investigated the socialization of filial piety attitudes, primarily using a cross-sectional design (e.g., Chen, 2014) or adult samples (e.g., Chen et al., 2016). Compared with adulthood, in which filial piety attitudes may already be quite formed, adolescence is a period of identity formation in which filial piety attitudes are still under development (Erikson, 1994; Hernández and Bámaca-Colbert, 2016). Additionally, teenagers may go through dramatic changes in their connections to their parents, including tendencies to challenge and redefine parental authority with their increasing needs for autonomy and independence, and may gain more exposure to new values and social experiences (McElhaney et al., 2009). It is thus important to understand the dynamics between youth’s filial piety attitudes and parenting, particularly during adolescence, using a longitudinal design.

### Effects of Parenting Styles on Filial Piety Attitudes

Filial piety is, to some extent, shaped through parental socialization, whereby children observe and acquire culturally sensitive schemas of parent–child interactions (Chen et al., 2016). During adolescence, despite the increasing influence of peer, parents remain an important socialization agent (Darling and Steinberg, 1993; Garcia et al., 2020). Through different parenting styles, parents create different socialization environments in which children perceive and understand what their parents desire from their intergenerational relationships, learn about how their parents expect to be treated by them, and gradually develop their filial piety attitudes. Parenting styles refer to constellations of parental attitudes and behaviors toward child, which creates a general emotional climate for parent–child interaction and parental socialization (Darling and Steinberg, 1993). Baumrind (1991) categorized parenting styles by two dimensions – responsiveness and demandingness. Responsiveness indicates the extent to which parents foster independence and self-regulation by attuning to their children’s specific needs, while demandingness indicates the extent to which parents request for children’s maturity and compliance. Authoritative (i.e., high responsiveness and demandingness) and authoritarian (i.e., low responsiveness but high demandingness) parenting styles represent two socialization climates that vary in parents’ responsiveness and demandingness (Baumrind, 1991; Darling and Steinberg, 1993), which are highly relevant to the socialization of filial piety (Chen, 2014). Authoritative parents are responsive and warm toward their children. Meanwhile, they have age-appropriate expectations and rules for their children and use reasonable strategies for discipline. They also grant their children freedom and autonomy to make decisions so as to develop their individuality and self-regulation. In contrast, authoritarian parents are cold or even hostile to their children and use high levels of control; they discipline their children harshly and demand unquestionable compliance without granting their children sufficient autonomy (Baumrind, 1991; Darling and Steinberg, 1993). Literature has suggested that authoritative parenting style is associated with desirable developmental outcomes in adolescents regardless of cultural backgrounds (e.g., Garcia et al., 2020; see a review, Wang and Chang, 2010), while authoritarian parenting style is associated with maladaptive outcomes in adolescents including Chinese ethnics (e.g., suicidal ideation: Lai and McBride-Chang, 2001; school performance: Pong et al., 2010).

The authoritative parenting style probably helps promote reciprocal filial piety attitudes. First, it can foster intimacy in the parent–child relationship, which is an affective building block of reciprocity. Through give-and-take with parents and experiences of warm parental involvement, children develop a relatively equalitarian relationship and an emotional bond with their parents, which enhance their mutuality and encourage their reciprocity. Previous research has revealed that the authoritative parenting style is related to higher quality of parent–child relationship (Chao, 2001). In addition, high levels of parental responsiveness and nurturing possibly foster children’s gratitude toward their parents, which motivates them to repay their parents through voluntary care and support. Indeed, Chen (2014) found that perceived authoritative parenting style was related to reciprocal filial piety among Hong Kong university students. Chen (2016) also found that adult children’s perceived supportive parenting (e.g., child-centeredness, positive reinforcement, and proactive teaching) was positively associated with their reciprocal filial piety 4 years later. Additionally, previous studies found that authoritative parenting style was positively associated with authoritarian filial piety (Chen, 2014) and that supportive parenting prospectively predicted authoritarian filial piety (Chen et al., 2016). According to self-determination theory (SDT; Ryan and Deci, 2000), people endorse cultural ideologies and practices with varying motivations. It is possible that one internalizes hierarchical values such as loyalty to a group or obedience to hierarchical relationships within one’s group out of personal choice (Chirkov et al., 2003). Therefore, such positive parenting can encourage children to endorse parental authority out of personal will. However, since there is no solid evidence showing an autonomous form of authoritarian filial piety in the literature, we explored the relationship between authoritative parenting and authoritarian filial piety attitudes in this study without a specific hypothesis.

Authoritarian parenting is unlikely to foster reciprocal filial piety, as it does not enable parents to build an affective bond with their children. Parents’ high demands for child obedience without appropriate reasoning and sufficient responsiveness might also deter children’s appreciation for their parents. Thus, authoritarian parenting may not be able to encourage reciprocal filial piety and may even dampen it. Chen (2014) did not find a significant association between authoritarian parenting and reciprocal filial piety. Rather, the authoritarian parenting style is likely to cultivate authoritarian filial piety. Chinese parents adopt authoritarian parenting, arguably out of a benign intention —“it is for your own good” (Chao, 1994; Camras et al., 2017). To train their children for a better future, Chinese parents adopt authoritarian parenting with an emphasis on parental authority and family solidarity, and they request children’s deference. Hence, such a parenting style readily conveys to children the importance of authoritarian filial piety. Ho (1994) showed that authoritarian filial piety was related to parents’ positive attitudes toward over-control and harshness. Chen (2014) also found that perceived authoritarian parenting style was positively related to authoritarian filial piety.

### Effects of Filial Piety Attitudes on Parenting Styles

While filial piety attitudes may be socialized by parents, children’s filial piety attitudes may likely also influence their parents’ parenting styles, according to the transactional model of socialization (Sameroff and Mackenzie, 2003). Indeed, much previous research has documented how parents act in response to children’s characteristics (e.g., Padilla-Walker et al., 2012; Moilanen et al., 2015). For instance, Lewis (1981) has contended that parents’ control may be an adaptation to children’s preexisting dispositions for compliance. Therefore, the socialization of filial piety among youth can be best understood by examining bidirectional influences between youth’s attitudes and parenting.

Filial piety attitudes reflect youth’s thoughts about how they should interact with their parents. These attitudes and the subsequent behavior of youth may provide feedback to their parents regarding whether their current parenting styles are proper or effective and, in turn, strengthen parents’ current parenting styles or lead parents to adjust their current styles. Adolescents with stronger reciprocal filial piety attitudes usually feel grateful of their parent’s love and care, and thus they will probably pay back through showing love and support to their parents and working hard to achieve parents’ expectations. The gratitude and good deeds of adolescents possibly encourage parents to show more warmth and support toward their children. Meanwhile, it also informs parents that it is not necessary to exert strict control over their children, and thus adolescents’ reciprocal filial piety will be associated with perceived parents’ decreased harshness and controlling behavior. In contrast, adolescents with stronger authoritarian filial piety attitudes usually show unquestionable obedience and reverence toward their parents out of parental authority, which informs parents that their current parenting style is legitimate and effective, regardless of which style they adopt. Thus, adolescents may perceive their parents to strengthen their existing parenting styles and show more corresponding parenting behavior. Briefly, adolescents’ reciprocal filial piety presumably encourages reciprocity of obligation and love in the parent–child relationship, whereas their authoritarian filial piety possibly strengthens parents’ original child-rearing style.

### Urban–Rural Variations

Filial piety is a notion originated from Confucianism in China, a representative cultural tradition of collectivism, which emphasizes relational hierarchies, including those in the parent–child relationship, and the fulfillment of social roles and obligations in such hierarchies (Ho, 1994; Schwartz et al., 2010). This differs from individualism, which emphasizes attainment of autonomy and personal goals (Hofstede, 2001). Going beyond the somewhat dichotomous and static approach to culture in terms of differentiating collectivism from individualism, Greenfield (2016) pointed out that cultural values evolve in broad socioeconomic contexts in an adaptive response to environmental demands, which in turn shape the socialization of children. She differentiated broad socioeconomic contexts into two prototypic social ecologies: *Gesellschaft* ecology, which refers to modern and primarily urban environments characteristic of complex economic systems, advanced technology, high average education levels, great diversity, and much contact with the outside world; and *Gemeinschaft* ecology, which refers to agriculturally based and primarily rural environments characteristic of simple labor division, low technology, low average education levels, little diversity, and limited contact with the outside world. *Gesellschaft* ecology fosters individualist values that underscore self-reliance, assertiveness, and autonomy, whereas *Gemeinschaft* ecology cultivates collectivist values that emphasize interdependence, obedience to authorities, and fulfillment of social duties, even at the cost of self-interests (Zeng and Greenfield, 2015). Social change theories (Kagitçibasi, 2007; Chen et al., 2015; Greenfield, 2016) have also argued that socioeconomic environments are dynamically changing in levels of economic development and modernization, thereby bringing about changes in cultural values and, in turn, the socialization of children. Since its open-up policies were initiated in the 1980s, China has gone through marked changes from a *Gemeinschaft* to *Gesellschaft* ecology, which manifested in increasing individualism (Xu and Hamamura, 2014; Zeng and Greenfield, 2015; see a review by Sun and Ryder, 2016). Notably, such a transformation is more salient in urban than rural areas due to the uneven levels of economic development and modernization between these areas. Extant research has indeed shown the resultant urban–rural differences in parental socialization and child development, with the parent–child relationship becoming more egalitarian in urban (vs. rural) families. For instance, urban parents demonstrate greater encouragement of initiative-taking toward their children than rural parents (Chen and Li, 2012). Meanwhile, urban adolescents feel less obligated to assist, respect, and support their family members (Fuligni and Zhang, 2004) and perceive it more acceptable to disagree openly with their parents than their rural counterparts (Zhang and Fuligni, 2006).

Parental socialization of filial piety may also be subject to the affordances of different social ecologies. In urban (vs. rural) areas with a heightened individualist orientation, youth may be more accepting of and responsive to authoritative parenting that satisfies their needs for independence and autonomy, but they are more likely to find authoritarian parenting aversive, as it thwarts their needs for independence and autonomy (Chen et al., 2015). As such, authoritative parenting may be more effective in urban (vs. rural) areas at fostering youth’s positive attitudes, such as filial piety, toward their parents, whereas authoritarian parenting may be less likely in urban (vs. rural) areas to foster, or even more likely to deter, the development of youth’s filial piety attitudes (especially reciprocal filial piety). In addition, the more equalitarian parent–child relationship in urban (vs. rural) areas may make urban (vs. rural) parents more sensitive to their children’s characteristics and more likely to adjust their parenting styles accordingly. Therefore, it can be expected that youth’s filial piety attitudes may be more predictive of parenting styles in urban (vs. rural) areas.

### Overview of the Current Study

Based on the dual filial piety model, and guided by the transactional model of socialization and change theories, the current study employed a two-wave (6 months apart) short-term longitudinal survey among early adolescents in urban and rural China to address three research questions. First, how may youth’s perceived parenting styles predict their filial piety attitudes over time? It was expected that, after adjusting for initial levels of filial piety at Time 1, perceived authoritative parenting at Time 1 would predict stronger reciprocal filial piety (Hypothesis 1) at Time 2, while perceived authoritarian parenting at Time 1 would predict weaker reciprocal filial piety (Hypothesis 2a) and stronger authoritarian filial piety (Hypothesis 2b) at Time 2. Second, how may youth’s filial piety attitudes predict their perceived parenting styles over time? It was expected that, after adjusting for initial levels of perceived parenting at Time 1, reciprocal filial piety at Time 1 would predict higher levels of perceived authoritative parenting (Hypothesis 3a) and lower levels of perceived authoritarian parenting (Hypothesis 3b) at Time 2, while authoritarian filial piety at Time 1 would predict higher levels of both perceived authoritative (Hypothesis 4a) and authoritarian parenting (Hypothesis 4b) at Time 2. Third, how may the aforementioned bidirectional associations between perceived parenting and youth’s filial piety attitudes vary between urban and rural areas in China? It was expected that the over-time positive links between youth’s perceived authoritative parenting and their reciprocal (Hypothesis 5a) would be stronger in the urban (vs. rural) area, as would the over-time negative link between youth’s authoritarian parenting and their reciprocal filial piety attitudes (Hypothesis 5b), while the over-time positive link between youth’s perceived authoritarian parenting and their authoritarian filial piety attitudes would be weaker in the urban (vs. rural) area (Hypothesis 5c). It was also expected that, generally, the over-time links between youth’s filial piety attitudes and their perceived parenting styles would be stronger in the urban (vs. rural) area (Hypothesis 6).

Notably, in the current study, which sampled early adolescents from urban and rural China, youth’s rather than parents’ reports on parenting styles were examined for two reasons. First, young adolescents are generally reliable reporters of their parents’ parenting behaviors, as they tend to be less biased than their parents, particularly in reporting harsh and controlling parental behaviors, including authoritarian parenting (Gonzales et al., 1996). Second, parenting behaviors as seen through the “eyes of the beholders” are conceptually meaningful (Steinberg et al., 1994; Barber, 1996) and have been widely found to influence youth’s developmental outcomes (e.g., Shek, 2007; Wang et al., 2007). For parsimony, the term “parenting styles” instead of youth’s “perceived parenting styles” was used when describing the results of the current study, while it is duly acknowledged that there may be differences in the findings based on adolescents’ versus parents’ reports on parenting (Pelegrina et al., 2003). It is also of note that a two-wave longitudinal design spanning 6 months was adopted, allowing for exploration of potential bidirectional effects between parenting styles and youth’s filial piety attitudes, which have rarely been investigated in previous research.

## Materials and Methods

### Participants and Procedure

A total of 850 secondary school students in China participated, including 314 urban students (155 boys and 159 girls) with a mean age of 13.31 years (*SD* = 0.36) and 536 rural students (293 boys and 243 girls) with a mean age of 13.72 years (*SD* = 0.55). The urban and rural samples did not differ significantly in gender composition [χ^2^_(1)_ = 2.232, *p* > 0.05], but the rural participants were generally older than the urban ones [*t*_(799)_ = 11.770, *p* < 0.001]. The urban students came from three schools serving middle and working classes in Shanghai, one of the most economically developed and modernized cities in China (see Liu et al., 2012). The per capita annual disposable income of urban residents was about USD$ 5,603.0 in Shanghai; 46.1% of the Shanghai residents received education of high school or above; 96.6% of the working population was engaged in second and tertiary industries (Shanghai Statistics Bureau, 2012). The cultural communication and economic collaboration with foreign countries were also frequent, manifested in the 4,329 new contracted project and 227 international exhibitions in 2011.

The rural students came from one school serving rural residents of Shantou in Guangdong Province. The per capita annual disposable income was about USD$ 1,204.0 in the rural area; 15.7% of the rural residents attained education level of high school or above (Shantou Statistics Bureau, 2012). The 23.1% of the rural working population were engaged in second industries due to the rapid growth of township industry, 57.8% in tertiary industries, while 24.2% of them remained in agriculture-related fields. Compared to Shanghai, the economic exchanges with foreign countries are less frequent, manifested in 46 new contracted projects with foreign companies in the whole Shantou district (including urban and rural areas) in 2011. Moreover, as shown in Table 1, on average, parental education levels were higher in the urban sample than in the rural sample [for mothers: *t*_(813)_ = 37.946, *p* < 0.001; for fathers: *t*_(821)_ = 31.334, *p* < 0.001]. The majority of the students lived in intact families, with the proportion being slightly greater in the rural (vs. urban) sample [χ^2^_(1)_ = 12.648, *p* < 0.001]. While the majority of the rural students had one or more siblings, the majority of the urban students were singletons [χ^2^_(1)_ = 611.920, *p* < 0.001], likely due to the one-child policy that has been administrated strictly in cities but loosely in the countryside (Chen et al., 2009).

**TABLE 1:** Demographic characteristics of the participants.

	Urban	(*N* = 314)	Rural	(*N* = 536)
	
	No.	Percentage	No.	Percentage
***Gender***				
Male	155	49.4%	293	54.7%
Female	159	50.6%	243	45.3%
***Mother’s education level***				
Primary education (1^a^)	7	2.2%	328	61.2%
Secondary education (2–3^a^)	113	36.0%	186	34.7%
Tertiary education (4–5^a^)	156	49.7%	2	0.4%
Postgraduate degree (6–7^a^)	23	7.3%	0	0.0%
No report	15	4.8%	20	3.8%
***Father’s education level***				
Primary education (1^a^)	5	1.6%	193	36.0%
Secondary education (2–3^a^)	116	36.9%	320	59.7%
Tertiary education (4–5^a^)	149	47.5%	8	1.5%
Postgraduate degree (6–7^a^)	32	10.2%	0	0.0%
No report	12	3.8%	15	2.8%
***Sibling status***				
Having one or more siblings	44	14.0%	519	96.8%
***Family type***				
Intact family	287	91.4%	513	95.7%

*The full sample was used in the data analysis. ^a^Mother’s/father’s education level: 1 = completion of primary school, 2 = completion of middle school, 3 = completion of high school; 4 = college-level sub-degree, 5 = bachelor’s degree, 6 = master’s degree, 7 = doctoral degree.*

The students completed a battery of questionnaires at two time points spanning 6 months, in the spring semester of grade 7 and the fall semester of grade 8. Among the 850 students who participated at Time 1, 263 urban students (136 girls and 127 boys) and 493 rural students (276 girls and 217 boys) participated at Time 2 as well, with attrition rates of 16.2% and 8.0%, respectively, due to students transferring to different schools, being absent on the day of the assessment, or losing interest. Attrition analysis was conducted to compare the differences in the study variables between students who participated at both times and those who participated at Time 1 only. Independent *t*-tests showed no differences in the urban sample. However, in the rural sample, the remaining participants reported lower levels of authoritarian parenting [*t*_(534)_ = −2.885, *p* < 0.01] and stronger reciprocal filial piety attitudes [*t*_(534)_ = 2.677, *p* < 0.01] than the non-remaining ones. At both times, the students completed the questionnaires during school hours in a self-administered manner with a trained research assistant or a teacher present in the classroom. School consent, parental consent, and students’ individual consent were obtained before the administration of the questionnaires. We had obtained ethics approval from the Survey and Behavioral Research Ethics Committee in the second author’s affiliated university before the implementation of the first wave of assessment.

### Measures

All measures were presented in Chinese and used a five-point Likert scale (1 = not at all true of me; 5 = very true of me). The means, standard deviations, internal reliability, and temporal stability of all measures are shown in Table 2.

**TABLE 2:** Descriptive statistics of the study variables.

	Urban							Rural					
	Time 1 (*n* = 314)			Time 2 (*n* = 263)			Time 1 (*n* = 536)				Time 2 (*n* = 493)		
Variables	*Mean*	*SD*	α	*Mean*	*SD*	α	*Mean*		*SD*	α	*Mean*	*SD*	α
Authoritative parenting	3.56	0.79	0.89	3.46	0.79	0.90	3.20		0.64	0.86	3.20	0.66	0.88
Authoritarian parenting	2.88	0.81	0.86	2.73	0.79	0.86	2.75		0.60	0.81	2.65	0.62	0.84
Reciprocal filial piety	4.02	0.71	0.89	4.04	0.76	0.91	3.86		0.65	0.86	3.90	0.65	0.87
Authoritarian filial piety	2.56	0.67	0.77	2.51	0.70	0.79	2.74		0.57	0.71	2.57	0.56	0.72

#### Filial Piety Attitudes

Students reported their attitudes toward different manifestations of filial piety using the Dual Filial Piety Scale (Yeh and Bedford, 2003), with eight items assessing reciprocal filial piety and eight items assessing authoritarian filial piety. Specifically, reciprocal filial piety taps into attitudes regarding support, care, and gratitude toward parents (e.g., “frequently concerned about my parents’ general well-being”), whereas authoritarian filial piety taps into attitudes toward unquestionable obedience toward parents as well as shouldering traditional obligations (e.g., “take my parents’ suggestions even when I do not agree with them”). We conducted a two-group confirmatory factor analysis (CFA) for the model with two latent factors representing reciprocal filial piety and authoritarian filial piety to test the measurement invariance across urban and rural samples. The results showed that metric equivalence was achieved between the urban and rural samples (CFI = 0.920; TLI = 0.913; RMSEA = 0.059), in which factor loadings were equal between the two groups (Kline, 2015). Compared to the configural model without any constraints, the model of metric equivalence showed similar model fit [Δχ^2^_(14)_ = 20.76, *p* > 0.05; ΔCFI = −0.002 < 0.01].

#### Parenting Styles

Students reported on their parents’ parenting styles using the Parental Authority Questionnaire (PAQ; Buri, 1991), with 10 items assessing authoritative parenting and 10 items assessing authoritarian parenting. We adopted a Chinese version of the PAQ that has been used with Chinese adolescents (McBride-Chang and Chang, 1998). A sample item for authoritative parenting would be “My parents have always encouraged verbal give-and-take whenever I have felt that family rules and restrictions were unreasonable,” while a sample item for authoritarian parenting would be “Whenever my parents told me to do something as I was growing up, they expected me to do it immediately without asking any questions.” Two-group CFA was conducted to test the measurement invariance between the urban and rural groups for the model with two latent factors representing authoritative parenting and authoritarian parenting. The results showed metric equivalence between the two groups [CFI = 0.885; TLI = 0.876; RMSEA = 0.064; Δχ^2^_(18)_ = 25.13, *p* > 0.05; ΔCFI = −0.001 < 0.01].

## Results

### Descriptive Analyses

As shown in Table 2, all measures were internally reliable in the current samples, and both filial piety attitudes and perceived parenting styles were quite stable over 6 months.

Four sets of multivariate analysis of variance were conducted to test urban–rural differences in the mean levels of filial piety attitudes and parenting styles at both times. At Time 1, urban students reported higher levels of reciprocal filial piety attitudes [*F*(1,848) = 11.366, *p* < 0.001, η_p_ = 0.013] and lower levels of authoritarian filial piety attitudes than their rural counterparts [*F*(1,848) = 18.755, *p* < 0.001, η_p_ = 0.022]. Yet the difference remained only in the reciprocal filial piety attitudes at Time 2 [*F*(1,754) = 6.659, *p* < 0.01, η_p_ = 0.009]. Additionally, at Time 1, urban students reported higher levels of authoritative parenting [*F*(1,847) = 52.527, *p* < 0.001, η_p_ = 0.058] and authoritarian parenting [*F*(1,847) = 7.359, *p* < 0.001, η_p_ = 0.007] than rural ones. Yet the difference remained only in the authoritative parenting at Time 2 [*F*(1,754) = 24.698, *p* < 0.001, η_p_ = 0.032].

### Cross-Lagged Path Analyses

**FIGURE 1:**
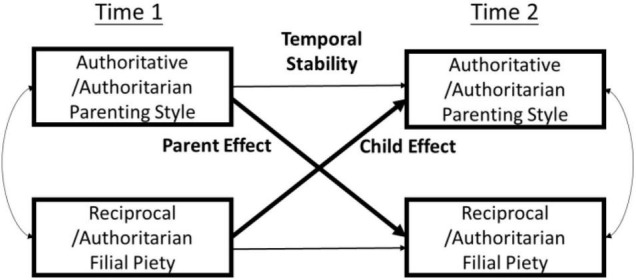
An illustration of the path analysis. Covariates (i.e., gender and parental education) and residuals are omitted for clarity of presentation.

The zero-order correlations among study variables are shown in Table 3. To explore potential transactional influences between parenting styles and students’ filial piety attitudes, a set of four cross-lagged path analyses examining over-time bidirectional associations between each perceived parenting style (i.e., authoritative or authoritarian parenting) and each type of attitude (i.e., reciprocal or authoritarian filial piety) was performed via AMOS 23.0 (Arbuckle, 2014). As shown in Figure 1, these analyses allow for stringent tests of the “parent effects” (indicated by the associations between Time 1 parenting and Time 2 filial piety) and the “child effects” (indicated by the associations between Time 1 filial piety and Time 2 parenting) simultaneously, while adjusting for the temporal stability of both parenting and filial piety (see Figure 1). We conducted separate analyses examining one parenting style vis-à-vis one type of filial piety attitudes. Compared with separate models, an integrative model with all study variables might yield unstable and misleading estimates due to multicollinearity of the parenting variables and the filial piety variables (see Stice and Barrera, 1995). An integrative model excludes the overlaps between the two parenting styles and the two types of filial piety attitudes, which might pose the problem of underestimating either the parent effects or the child effects, especially when the effects were expected to be small in a longitudinal design that strictly controls for temporal stability of the variables. Second, partialling out the overlaps may make the urban–rural differences obscured in the integrative model, because the urban–rural differences may partially rest in the overlaps (see Wang et al., 2007). Therefore, we opted for separated models in this study. Additionally, given that parental education (e.g., Xu et al., 2005; Li et al., 2014) and child gender (e.g., Wong et al., 2010; Xia, 2020) have been found to be related to parenting styles and youth’s filial piety attitudes, they were included as covariates in the path analyses. Missing data were handled by full information maximum likelihood (FIML), as this method has been found to outperform other *ad hoc* methods, such as listwise or pairwise deletion or mean imputation, for handling missing data (Byrne, 2013). FIML has been frequently used to address the issue of attrition in longitudinal studies with repeated measure (e.g., Van Ouytsel et al., 2019; Salmela-Aro et al., 2021). To identify potential urban–rural variations in the parent effects and child effects, two-group path analyses were performed by comparing pairs of models—an unconstrained model where the aforementioned paths were freely estimated for the urban and rural samples and a constrained model where these paths were set to be equal in the two samples. Each model was evaluated in terms of model fit as indicated by the Comparative Fit Index (CFI) and the Tucker and Lewis’s Index (TLI), with values greater than 0.95 suggesting a good fit and values greater than 0.90 but smaller than 0.95 suggesting adequate fit, and by the root mean square error of approximation (RMSEA), with values smaller than 0.03 indicating a good fit and values smaller than 0.06 but greater than 0.03 suggesting an adequate fit (Kline, 2015). In addition, χ^2^ difference tests were conducted to compare pairs of unconstrained and constrained models. Specifically, a significant χ^2^ difference between an unconstrained and a constrained model would indicate that the parent effect or the child effect under examination was different between the urban and rural samples; otherwise, the path would be deemed similar in the two samples, and the constrained model would be reported as the final model. Table 4 shows the estimated path coefficients and the model fit for the final models. All final models fit the data well (CFIs > 0.99; TLIs > 0.95; RMSEAs < 0.03).

**TABLE 3:** Bivariate correlations among the study variables.

Variables	1	2	3	4	5	6	7	8	9	10
***Time 1***										
Authoritative parenting	–	−0.31***	0.47***	0.23***	0.68***	−0.43***	0.49***	0.21***	–0.04	0.23***
Authoritarian parenting	–0.05	–	−0.16***	–0.01	−0.38***	0.59***	−0.23***	–0.003	0.25***	0.05
Reciprocal filial piety	0.42***	–0.04	–	−0.46***	0.40***	−0.32***	0.62***	0.30***	–0.04	0.08
Authoritarian filial piety	0.26***	0.24***	0.41***	–	0.20**	–0.07	0.22***	0.59***	0.21***	0.10
***Time 2***										
Authoritative parenting	0.59***	−0.25***	0.35***	0.22***	–	−0.55***	0.57***	0.26***	–0.03	0.17**
Authoritarian parenting	−0.17***	0.61***	−0.12*	0.14***	−0.34**	–	−0.28***	–0.01	0.16*	–0.08
Reciprocal filial piety	0.32***	0.01	0.53***	0.27***	0.46***	−0.11*	–	0.37***	–0.09	0.11
Authoritarian filial piety	0.11*	0.17***	0.24***	0.53***	0.21***	0.22***	0.36***	–	0.16*	0.07
Gender	–0.02	0.16***	−0.13***	0.17***	0.02	0.14**	−0.13**	0.18***	–	0.00
Parents’ education	0.08	0.04	–0.01	–0.02	0.05	0.08	0.01	0.08	0.05	–

Correlations for the urban sample are above the diagonal, and those for the rural sample are below the diagonal. Gender: 0 = female, 1 = male; parents’ education was indexed by an average score of father’s and mother’s education levels, and their correlations (r) were 0.62*** and 0.26* for urban and rural samples, respectively.

*p < 0.05; **p < 0.01; ***p < 0.001.

**TABLE 4:** Estimates of the parent effects and the child effects in the path analyses.

	Urban			Rural			Model	Fit		
	Unstd.	SE	Std.	Unstd.	SE	Std.	Δχ^2^_(1)_	CFI	TLI	RMSEA
**Bidirectional associations between Authoritative PS and RFPA**								**0.998**	**0.987**	**0.019**
Parent effect: Authoritative PS → RFPA	0.171***	0.033	0.184	0.171***	0.033	0.163	3.285			
Child effect: RFPA → Authoritative PS	0.131***	0.033	0.121	0.131***	0.033	0.127	0.025			
Temporal stability: Authoritative PS	0.583***	0.032	0.594	0.583***	0.032	0.558	0.676			
Temporal stability: RFPA	0.501***	0.034	0.491	0.501***	0.034	0.486	0.534			
**Bidirectional associations between Authoritarian PS and RFPA**								**0.997**	**0.968**	**0.025**
Parent effect: Authoritarian PS → RFPA	−0.096*	0.046	–0.107	0.063	0.042	0.057	4.807*			
Child effect: RFPA → Authoritarian PS	−0.195***	0.053	–0.173	−0.078*	0.034	–0.083	4.733*			
Temporal stability: Authoritarian PS	0.589***	0.029	0.594	0.589***	0.029	0.581	2.508			
Temporal stability: RFPA	0.567***	0.046	0.551	0.567***	0.046	0.556	2.253			
**Bidirectional associations between Authoritative PS and AFPA**								**0.999**	**0.993**	**0.012**
Parent effect: Authoritative PS → AFPA	0.009	0.028	0.010	0.009	0.028	0.010	2.738			
Child effect: AFPA → Authoritative PS	0.077*	0.035	0.066	0.077*	0.035	0.065	0.045			
Temporal stability: Authoritative PS	0.623***	0.030	0.634	0.623***	0.030	0.596	0.356			
Temporal stability: AFPA	0.552***	0.032	0.643	0.552***	0.032	0.010	0.302			
**Bidirectional associations between Authoritarian PS and AFPA^#^**								**1.000^#^**	**1.002^#^**	**0.020**
Parent effect: Authoritarian PS → AFPA	0.022	0.028	0.027	0.022	0.028	0.023	0.237			
Child effect: AFPA → Authoritarian PS	–0.004	0.033	–0.003	–0.004	0.033	–0.004	0.601			
Temporal stability: Authoritarian PS	0.606***	0.030	0.615	0.606***	0.030	0.593	0.939			
Temporal stability: AFPA	0.551***	0.031	0.542	0.551***	0.031	0.543	2.574			

This table shows the coefficients of final model with some paths constrained to be equal between the urban and rural settings. Δχ^2^ indicates chi-square change by constraining the structural path to be equal in urban and rural settings, compared with the unconstrained model. CFI, TLI, and RMSEA represent the model fit of the final model. Gender and parental education were included as covariates in the models. ^#^The CFI and TLI were equal to or larger than 1 because the model was just-identified; since the focus was on the relationships between the variables (i.e., the parent effect and the child effect), the fit was not of concern (see Valenzuela and Bachmann, 2017). *p < 0.05; ***p < 0.001. PS, parenting style; RFPA, reciprocal filial piety attitudes; AFPA, authoritarian filial piety attitudes.

#### Parenting Styles and Reciprocal Filial Piety Attitudes

The final model examining authoritative parenting vis-à-vis reciprocal filial piety contained equal parental and child effects. This constrained model did not differ from the unconstrained model in model fit [Δχ^2^_(4)_ = 6.406, *p* > 0.05]. As shown in Table 4, there was a significant parent effect in both the urban and rural samples, such that authoritative parenting predicted students’ increased reciprocal filial piety 6 months later (supporting Hypothesis 1), with this effect being of similar strength in the two samples (not supporting Hypothesis 5a). There was also a significant child effect in both the urban and rural samples, such that students’ reciprocal filial piety predicted increased authoritative parenting 6 months later (supporting Hypothesis 3a), with this effect being of similar strength in the two samples (not supporting Hypothesis 6). The final model examining authoritarian parenting vis-à-vis reciprocal filial piety contained unequal child and parental effects [Δχ^2^_(2)_ = 4.730, *p* > 0.05]. Only in the urban sample was there a significant parent effect, such that authoritarian parenting predicted decreased reciprocal filial piety 6 months later (supporting Hypotheses 2a and 5b), while there was a significant child effect in both the urban and rural samples, such that students’ reciprocal filial piety predicted decreased authoritarian parenting 6 months later, with this effect being stronger in the urban (vs. rural) sample (supporting Hypotheses 3b and 6).

#### Parenting Styles and Authoritarian Filial Piety Attitudes

The final model examining authoritative parenting vis-à-vis authoritarian filial piety contained equal parent and child effects [Δχ^2^_(4)_ = 5.374, *p* > 0.05]. There was no significant parent effect in either the urban sample or the rural sample, such that authoritative parenting at Time 1 was unrelated to authoritarian filial piety at Time 2, after adjusting for authoritarian filial piety at Time 1 (not supporting Hypothesis 2b). Meanwhile, there was a significant child effect in both the urban and rural samples, such that students’ authoritarian filial piety predicted increased authoritative parenting 6 months later (supporting Hypothesis 4a), with this effect being of similar strength in the two samples (not supporting Hypothesis 6). The final model examining authoritarian parenting vis-à-vis authoritarian filial piety contained equal parent and child effects [Δχ^2^_(4)_ = 4.446, *p* > 0.05]. In both the urban and rural samples, there was neither significant parent effect nor child effect, such that authoritarian parenting and authoritarian filial piety were unrelated to each other over time, after adjusting for their temporal stabilities (not supporting Hypotheses 2b and 4b).

## Discussion

Going beyond previous research that has mainly focused on the effects of parenting on filial piety in a single social ecology (e.g., Chen, 2014), this study examined the bidirectional associations between perceived parenting and filial piety attitudes in a two-wave longitudinal study spanning 6 months and compared these associations between urban and rural Chinese early adolescents. As expected, over time, perceived authoritative parenting predicted increased reciprocal filial piety among both urban and rural adolescents, whereas perceived authoritarian parenting predicted decreased reciprocal filial piety among urban adolescents only. Moreover, in both urban and rural areas, reciprocal filial piety predicted heightened perceived authoritative parenting and lessened perceived authoritarian parenting 6 months later, with the latter effect being stronger among urban adolescents. However, there was only one significant over-time association between perceived parenting styles and authoritarian filial piety, such that, in both the urban and rural areas, authoritarian filial piety predicted heightened perceived authoritative parenting 6 months later. The findings highlight transactional influences between parents and youth in the socialization process (Sameroff and Mackenzie, 2003) and testify to the role of socioeconomic and sociocultural changes in shaping parental socialization and youth development (Kagitçibasi, 2007; Chen et al., 2015; Greenfield, 2016).

### Transactions Between Parenting Styles and Filial Piety Attitudes

Adding to mounting evidence that parents are still influential in child development during adolescence (e.g., Wang et al., 2007; Chen, 2014), the current findings suggest that parenting styles affect the development of filial piety attitudes among early adolescents in China, even though this is during an age when youth increasingly strive for self-reliance and individuality. The findings show that, when youth perceived their parents to be authoritative (i.e., warm, responsive, and nurturing toward them), they were more likely to develop reciprocal filial piety attitudes over time, probably because such parenting satisfies youth’s needs for independence and autonomy and may be better accepted and appreciated, thus more likely eliciting youth’s reciprocal respect and care for their parents. In contrast, when youth perceived their parents to be authoritarian (i.e., cold and controlling), they were less likely to develop reciprocal filial piety over time, probably because such parenting thwarts youth’s needs for independence and autonomy and may be aversive to youth, thus alienating them from feeling grateful and caring toward their parents. Furthermore, moving beyond extant research, the findings present first-time evidence on the potential effects of youth’s filial piety attitudes on parenting. When youth endorsed reciprocal filial piety, over time, they were more likely to perceive increased use of authoritative parenting but decreased use of authoritarian parenting by their parents. This may be because parents sense and appreciate the youth’s reciprocal filial piety attitudes through their respect and caring for their parents as well as their willingness to follow their parents’ wishes, which, in turn, may lead parents to become warmer and more nurturing (i.e., adopting authoritative parenting) rather than harsher and more controlling (i.e., adopting authoritarian parenting) toward the youth.

The aforementioned findings of over-time bidirectional associations between parenting and youth’s filial piety attitudes lend further support for the transactional model of socialization (Sameroff and Mackenzie, 2003) and, more generally, for systems perspectives on human development that highlight the active role of youth in shaping their own developmental niches (e.g., Bronfenbrenner and Morris, 1998; Lerner et al., 2015). The family is a system in which multiple members interact, and the child’s role should not be downplayed (Cox and Paley, 1997). As Kerr et al. (2012) argued, “parenting is in part a reaction to adolescent behavior” (p. 1550). This study indeed showcases such dynamic mutual influences between parents and youth in the development of filial piety.

In fact, the findings are particularly informative for a nuanced understanding of the Dual Filial Piety Model. Parents may believe that authoritarian parenting, which emphasizes absolute parental authority, would be effective at fostering filial piety among children in terms of unquestionable reverence and obedience, as parents who want their children to develop filial piety are more likely to use authoritarian parenting (Pearson and Rao, 2003; Rao et al., 2003). However, inconsistent with such expectations, the current study found that youth’s perceived authoritarian parenting did not predict their authoritarian filial piety over time. It is possible that parents’ simple use of authoritarian parenting does not get across to children their agenda to foster filial piety. Future research on potential antecedents of authoritarian filial piety could investigate more explicit and direct parental socialization attempts targeting filial piety, such as communication of parental expectations specifically regarding filial piety (e.g., “My father expects me to have good behavior so that I will not bring dishonor to the family”; Shek, 2007). The current study also found that, when youth endorsed authoritarian filial piety, over time, they were more likely to perceive increased use of authoritative parenting by their parents. This is consistent with previous research showing that, when children demonstrated proper attitudes and behavior, parents used more authoritative parenting (e.g., Moilanen et al., 2015).

Taken together, the different patterns of the over-time bidirectional associations of reciprocal versus authoritarian filial piety with parenting styles reaffirm the importance of differentiating these two types of filial piety. Key to their distinction from each other is the degree to which they are compatible with youth’s need for autonomy (i.e., striving to make decisions for oneself and being in control of one’s own important life affairs; Ryan and Deci, 2000). Researchers and practitioners as well as parents should be aware of the differences between filial piety that results from mutual love and care (i.e., reciprocal filial piety) and filial piety indoctrinated through dogma and authority (i.e., authoritarian filial piety), including their distinct socialization processes and adjustment outcomes. Filial piety, though originated in Confucian ethics, may represent a universal psychological schema of the parent–children relationship (Bedford and Yeh, 2021) that bears broad and profound implications for research on intergenerational relationships beyond the Chinese context. As argued by Bedford and Yeh (2019), filial piety encompasses two fundamental psychological needs: the need for interpersonal relatedness and the need for collective identity in the context of the intergenerational relationship. The dual filial piety model relates these two needs to filial norms that may vary across cultures by attending to another fundamental psychological need—the need for autonomy. Notions that share a common element of family primacy but have been conceptualized in research with other ethnic and cultural groups and are based on other ethnic and cultural ethos (e.g., family obligation, see Fuligni and Pedersen, 2002; familism, see Padilla et al., 2016) may also be guided by either reciprocity, authoritarianism, or both and, hence, correspond to reciprocal and/or authoritarian filial piety. The dual filial piety model is thus of great heuristic value for research examining the parent–child relationship across cultures. For instance, emotional reciprocity may be a particular focus of study in the affection-based parent–child relationship typically upheld in individualist cultures.

### Urban–Rural Variations

When it comes to urban–rural variations, the current study found both similarities and differences in the over-time bidirectional associations between parenting style and filial piety. Among both urban and rural adolescents, perceived authoritative parenting predicted stronger reciprocal filial piety over time, while both reciprocal and authoritarian filial piety predicted higher levels of perceived authoritative parenting over time. These urban–rural similarities support the views that the growing need for autonomy during adolescence (Helwig, 2006) and the transactional nature of socialization (Sameroff and Mackenzie, 2003) may be universal developmental processes. Self-determination theory (Ryan and Deci, 2000) argues that people across cultures have the basic psychological need for autonomy, and this need becomes increasingly salient during adolescence. Much previous research has documented that, regardless of the sociocultural context where adolescents reside, parenting that nurtures their autonomy tends to be well received and appreciated, whereas parenting that dampens their autonomy may be detrimental, which is consistent with the current findings of the positive transactions between authoritative parenting and reciprocal filial piety. Notably, there has been a concern that socioeconomic development and modernization might lead to the decay of filial piety and other family values in societies (Aboderin, 2004; Cheung and Kwan, 2009). Nonetheless, the current findings indicate that filial piety based on reciprocity and parenting characterized by warmth and the allowance of autonomy may well mutually facilitate each other in not only a traditional rural area but also a highly modernized urban area in contemporary China.

In terms of urban–rural differences, the current study found that only among urban adolescents was perceived authoritarian parenting a predictor of weaker reciprocal filial piety over time, while perceived authoritarian parenting was unrelated to reciprocal filial piety over time among rural adolescents. Moreover, while reciprocal filial piety predicted lower levels of perceived authoritarian parenting over time in both the urban and rural samples, this effect was stronger among urban (vs. rural) adolescents. These findings suggest a greater need for autonomy among urban (vs. rural) adolescents in that authoritarian parenting, which thwarts this need, is more likely to alienate them from their parents than is the case for rural adolescents. This greater need for autonomy may, in large part, have resulted from socioeconomic development in the cities toward a market-oriented economy, which demands independent workers who are good at self-expression and self-governance. Greater exposure in the cities (vs. the countryside) to Western ideologies that stress the self as a separate and distinct entity from others (Kagitçibasi, 2007; Chen and Li, 2012) may also have contributed to heightened value placed on independence and autonomy that is shared by socialization influences on them (e.g., peers and social media; Arnett, 1995; Chen et al., 2015) other than their parents. With a greater need for and heightened value placed on independence and autonomy, urban (vs. rural) adolescents may be more likely to find authoritarian parenting aversive, resulting in the finding that perceived authoritarian parenting predicted weaker reciprocal filial piety among urban (but not rural) adolescents. In addition, in line with previous research (e.g., Zhang and Fuligni, 2006; Chen and Li, 2012), the current findings suggest a more equalitarian parent–child relationship in urban (vs. rural) China in that the over-time link from reciprocal filial piety to lower levels of perceived authoritarian parenting was stronger among urban (vs. rural) adolescents. Socioeconomic development and exposure to Western ideologies in the cities may have transformed not only youth’s views and values but also parents’ socialization goals and practices (e.g., heightened emotional value emphasizing enjoyment in the parent–child relationship, and lessened instrumental value in terms of expecting financial returns from children in parents’ old age). With such transformed values, urban (vs. rural) parents may more readily adjust their parenting styles (especially changing a style that is contrary to an egalitarian parent–child relationship) to their children’s characteristics, thus resulting in a stronger association of adolescents’ reciprocal filial piety with lower levels of perceived authoritarian parenting over time in the urban (vs. rural) sample.

### Limitations and Future Directions

Despite its notable contributions in revealing the dynamic socialization and developmental processes of dual filial piety and the role of social ecologies in shaping these processes, the current study had a number of limitations, pointing toward directions for future research. First, the current study relied solely on adolescent self-reports. Previous studies have suggested that there are discrepancies between parent reports and adolescent reports on family processes (e.g., De Los Reyes et al., 2016). More importantly, parent-reported parenting and adolescent-reported parenting may have dissimilar impacts on adolescent adjustment (e.g., Pelegrina et al., 2003; Chen et al., 2016). The question remains whether parent reports on parenting may yield different findings from the current ones based on adolescent reports, and future studies would benefit from including multiple informants regarding parenting. Second, the current study employed stringent longitudinal analyses that adjusted for temporal stabilities of parenting and filial piety and is thus more informative regarding the direction of effects between parenting and filial piety than previous research that has not adjusted for temporal stabilities (e.g., Chen et al., 2016). However, caution is still warranted in drawing causal conclusions and replication from future research. Third, the current study examined parenting by addressing both parents as one unit instead of assessing maternal and paternal parenting as well as youth’s filial piety attitudes toward mothers and fathers separately. Some preliminary findings have shown that Hong Kong school-aged children have reported stronger filial piety attitudes toward their mother than father, while maternal and paternal warmth and traditional Chinese parenting (e.g., expecting children to be obedient) related similarly to children’s general filial piety attitudes (Lin and Yip, 2011). Future research examining mother–child dyads and father–child dyads separately could provide a refined picture of the socialization processes of filial piety. Fourth, the current urban and rural samples were each recruited from one site only and are not representative of vast urban and rural areas in China, thus limiting the generalizability of the findings. Despite salient socioeconomic differences between the two sites chosen for the current study, the rural sample was from a relatively less underdeveloped area in the countryside of China with its average per capita net income (i.e., USD$ 1,220.7) higher than the national average of rural districts (i.e., USD$ 1,079; National Bureau of Statistics of the P.R. China, 2012). More and larger urban–rural differences than those documented in the current study may be observed between urban areas and highly underdeveloped rural area. Additionally, the current sample size for each site was limited, which influences the statistic power to detect small longitudinal effects. Therefore, future studies are needed to investigate multiple regions with different levels of socioeconomic development and recruit a larger sample (see Chen and Li, 2012). Moreover, the urban and rural samples were recruited from different parts of China (i.e., Shanghai in the east coast vs. Guangdong Province in the south-east coast), and thus we could not exclude the possibility that the observed differences were due at least in part to subcultural variations between these two geographically distant parts of the country. Yet, the urban sample from Shanghai and the rural sample from rural Guangdong did capture well the contrast between the Gesellschaft (urban) ecology and the Gemeinschaft (rural) ecology in terms of economic and technological developments, education levels, diversity and contact with the outside world (Greenfield, 2016). Future research better teasing apart subcultural versus urban–rural variations is needed (e.g., comparing urban and rural samples from the same province). Lastly, although the current study was well guided by social change theories to examine urban–rural variations and revealed meaningful similarities and differences in the urban and rural samples, it did not directly investigate the psychological needs (e.g., youth’s need for autonomy) and values (e.g., independence and an egalitarian parent–child relationship) that are assumed to underlie urban–rural variations. Future research is needed to unpack the features and mechanisms through which urban and rural ecologies shape the socialization and development of filial piety among Chinese adolescents.

## Conclusion

To our knowledge, this is the first study to examine the transactions between parenting styles and filial piety attitudes and compare these transactions between urban and rural ecologies. In particular, the findings from this study support the dual filial piety model as a guiding framework for research on filial piety across cultures, and they contribute to an advanced understanding of dual filial piety by extending previous research to explore transactional socialization dynamics between parents and youth as well as urban–rural variations. More broadly, the findings bear implications for theories and practices concerning the socialization and development of psychological constructs that are generally crucial in the parent–child relationship across cultures, suggesting that researchers and practitioners in psychological counseling and therapy and other helping professions pay attention to children’s active role in the construction of the family environment as well as to the role of socioeconomic and sociocultural factors in the way a family functions.

## Data Availability Statement

The raw data supporting the conclusions of this article will be made available by the authors, without undue reservation.

## Ethics Statement

The studies involving human participants were reviewed and approved by Survey and Behavioral Research Ethics Committee of The Chinese University of Hong Kong. Written informed consent to participate in this study was provided by the participants’ legal guardian/next of kin.

## Author Contributions

LL designed the study, analyzed and interpreted the data, and drafted the manuscript. QW co-designed the study, participated in the interpretation of results, and performed professional editing throughout the manuscript. Both authors contributed to the article and approved the submitted version.

## Conflict of Interest

The authors declare that the research was conducted in the absence of any commercial or financial relationships that could be construed as a potential conflict of interest.

## Publisher’s Note

All claims expressed in this article are solely those of the authors and do not necessarily represent those of their affiliated organizations, or those of the publisher, the editors and the reviewers. Any product that may be evaluated in this article, or claim that may be made by its manufacturer, is not guaranteed or endorsed by the publisher.
